# Multiple Roles of Vestigial-Like Family Members in Tumor Development

**DOI:** 10.3389/fonc.2020.01266

**Published:** 2020-07-24

**Authors:** Noritaka Yamaguchi

**Affiliations:** ^1^Laboratory of Molecular Cell Biology, Graduate School of Pharmaceutical Sciences, Chiba University, Chiba, Japan; ^2^Department of Molecular Cardiovascular Pharmacology, Graduate School of Pharmaceutical Sciences, Chiba University, Chiba, Japan

**Keywords:** VGLL, vestigial, TEAD, Hippo, YAP, TAZ

## Abstract

Vestigial-like family (VGLL) members are mammalian orthologs of *vestigial* gene in *Drosophila*, and they consist of four homologs (VGLL1–4). VGLL members have TDU motifs that are binding regions to TEA/ATSS-DNA-binding domain transcription factor (TEAD). Through TDU motifs, VGLL members act as transcriptional cofactors for TEAD. VGLL1-3 have single TDU motif, whereas VGLL4 has two tandem TDU motifs, suggesting that VGLL4 has distinct molecular functions among this family. Although molecular and physiological functions of VGLL members are still obscure, emerging evidence has shown that these members are involved in tumor development. Gene alterations and elevated expression of *VGLL1-3* were observed in various types of tumors, and VGLL1-3 have been shown to possess tumorigenic functions. In contrast, down-regulation of VGLL4 was detected in various tumors, and the tumor-suppressing role of VGLL4 has been demonstrated. In this review, we summarize the recently identified multiple roles of VGLL members in tumor development and provide important and novel insights regarding tumorigenesis.

## Introduction

**Figure 1:**
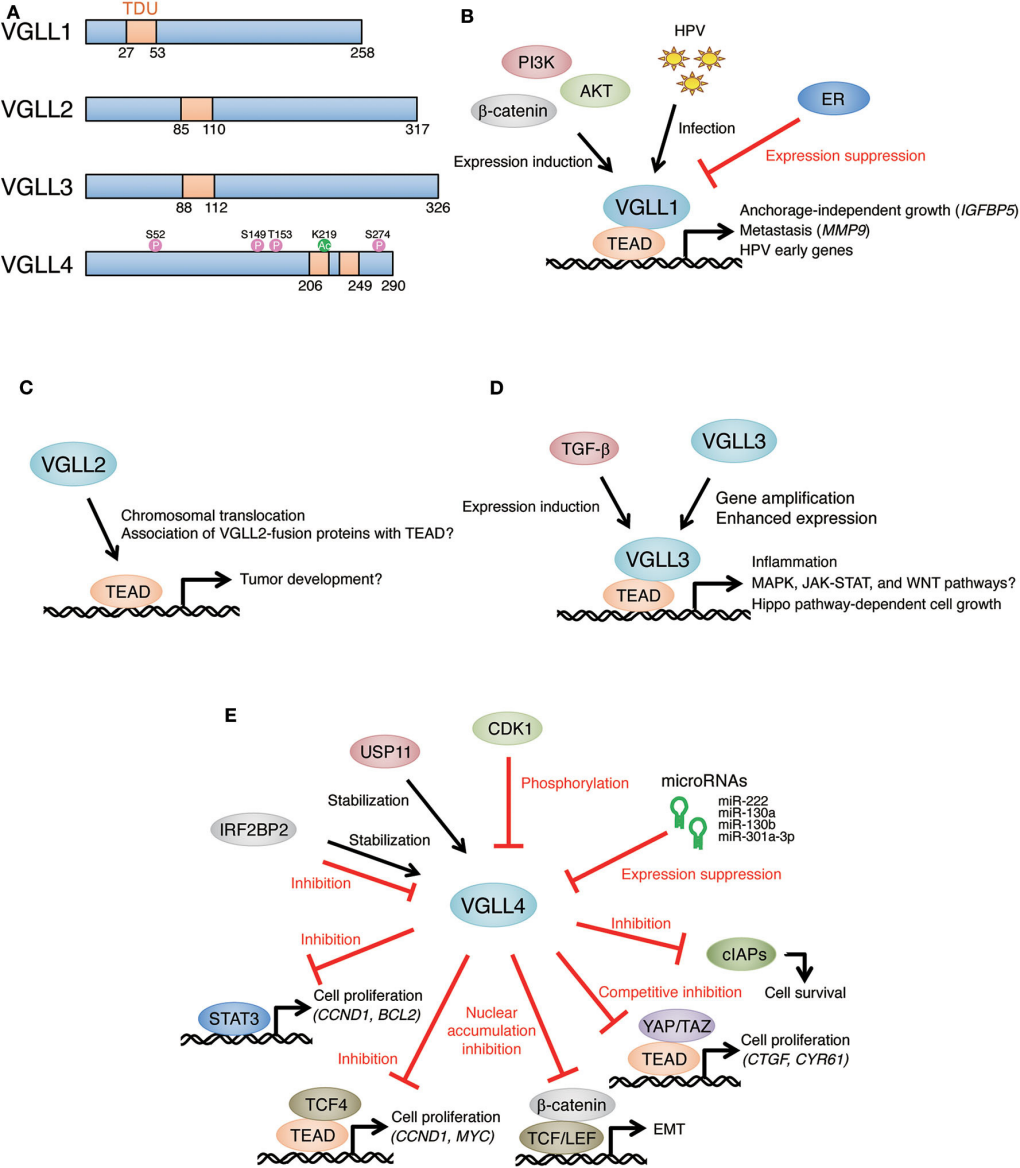
Multiple roles of VGLL members in tumor development. **(A)** Schematic representations of VGLL family proteins. Domain architecture is based on (7). CDK1-mediated phosphorylation sites (8) and a p300-mediated acetylation site (9) are shown on the VGLL4 structure. TDU, Tondu motif. **(B)** Overexpression of VGLL1 promotes anchorage-independent growth of prostate tumor cell lines through the induction of IGFBP5. VGLL1 expression was increased by the PI3K-AKT-β-catenin pathway and induced metastasis via MMP9 expression in gastric tumor. VGLL1 was induced by HPV infection, and VGLL1 contributed to HPV early gene expression. Estrogen receptor (ER) repressed VGLL1 expression in breast tumor. **(C)** *VGLL2* fusion genes (*VGLL2-CITED2* and *VGLL2-NCOA2*) were identified in pediatric spindle and sclerosing rhabdomyosarcoma. Although the tumorigenicity of these genes is obscure, it has been suggested that these genes affect gene expression signatures in tumor. **(D)** *VGLL3* gene amplification was detected in myxoinflammatory fibroblastic sarcoma and soft tissue sarcoma, and VGLL3 was involved in the proliferation of sarcoma cells. VGLL3 expression showed positive correlation with poor prognosis in gastric tumor patients, and the activation of MAPK, JAK-STAT, WNT pathways as well as enhanced immune infiltrates were observed in VGLL3-high tumor. VGLL3 expression was induced by TGF-β-stimulation in a histone-modification dependent manner. **(E)** VGLL4 as a tumor suppressor through competition with YAP/TAZ for TEAD-binding in various tumors. Inactivation of VGLL4 was caused by microRNA-mediated gene silencing or CDK1-mediated phosphorylation. USP11 stabilized VGLL4 and enhanced its tumor-suppressing role. IRF2BP2 had both positive and negative effects on VGLL4. TCF4, STAT3, β-catenin, and cIAPs are also targets of VGLL4-mediated suppression.

Vestigial-like family (VGLL) is composed of four homologous members (VGLL1, 2, 3, and 4) in mammals (1, 2). *VGLL* genes are orthologs of *vestigial* (*vg*) which was primarily identified as a gene required for wing development in *Drosophila* (3, 4). Vg binds to the product of *scalloped* (*sd*), which belongs to a conserved transcription factor family having a TEA/ATSS-DNA-binding domain (TEAD). It lacks DNA-binding domains and binds to DNA as a Vg-Sd protein complex. Vg acts as a cofactor for Sd, and Vg-Sd complex regulates the expression of genes involved in wing development (5). Furthermore, Vg has a short motif comprising ~26 amino acids, which is required and sufficient for its association with Sd. This motif is conserved in mammalian VGLL members (1, 2). VGLL1 was the first isolated mammalian VGLL member with structural and functional similarity to Vg (6). VGLL1 was originally named TONDU (TDU), and therefore the motif in VGLL members required for association with TEAD is known as the TDU motif. VGLL1-3 have a single TDU motif, whereas VGLL4 has two separated TDU motifs (Figure 1A), suggesting that VGLL1-3 and VGLL4 have distinct molecular functions (1, 2).

Mammalian TEAD family consist of four homologs (TEAD1, 2, 3, and 4). Studies so far have revealed that the dysregulation of TEAD activity causes tumorigenesis (10–12). TEAD activity is controlled by the Hippo tumor-suppressor pathway. The main targets of the Hippo pathway are two homologous transcriptional cofactors for TEAD: Yes-associated protein (YAP) and transcriptional cofactor with PDZ-binding motif (TAZ) (13, 14). The Hippo pathway promotes protein degradation and nuclear export of YAP/TAZ through phosphorylation by protein kinases large tumor suppressor 1/2 (LATS1/2) (15). YAP/TAZ-TEAD complex promotes the expression of genes involved in cell growth, and disruption in the Hippo pathway causes aberrant activation of this complex and thereby induces tumor development (10–12).

Given that VGLL is a cofactor family for TEAD, VGLL members are likely to be involved in tumorigenesis (16, 17). Emerging evidence has revealed that these proteins have both promoting and suppressive roles in tumor development. The aim of this review is to summarize the multiple roles of VGLL members in tumor development (Table 1).

**Table 1:** Summary of the involvement of VGLL members in various types of tumors.

	**Tumor types**	**Effects on tumor**	**Important feature**	**References**
VGLL1	Breast tumor	Association with reduced overall survival	Increased expression of VGLL1	(18)
	Cervical tumor	Tumor development	VGLL1-mediated HPV early gene expression	(19)
	Gastric tumor	Enhancement of cell proliferation and metastasis	Positive correlation between VGLL1 and PI3K/AKT/β-catenin signaling	(20)
	Pediatric neuroepithelial neoplasm	Oncogene?	*EWSR1-VGLL1* fusion gene	(21)
	Prostate tumor	Enhancement of anchorage-independent growth	VGLL1-mediated IGFBP5 expression	(22)
	Soft tissue malignant myoepithelial tumor	Oncogene?	*EWSR1-VGLL1* fusion gene	(23)
VGLL2	Pediatric spindle and sclerosing rhabdomyosarcoma	Oncogene?	*VGLL2-CITED2* and *VGLL2-NCOA2* fusion genes	(24)
VGLL3	Breast tumor and sarcoma	Enhancement of cell proliferation	Hippo pathway-dependent cell proliferation	(25)
	Gastric tumor	Positive correlation with poor prognosis	Positive correlation between VGLL3 and MAPK/JAK-STAT/WNT signaling	(26)
	Myxoinflammatory fibroblastic sarcoma	Oncogene?	*VGLL3* gene amplification	(27)
	Ovarian tumor	Tumor suppressor gene?	Tumor suppressive role of chromosome fragments containing *VGLL3*	(28)
	Soft tissue sarcoma	Enhancement of cell proliferation	*VGLL3* gene amplification	(29)
VGLL4	Bladder tumor	Repression of cell proliferation and invasion	miR-130b-mediated VGLL4 repression	(30)
	Breast tumor	Repression of cell proliferation	VGLL4-mediated iInhibition of YAP-TEADs	(31)
	Breast tumor	Repression of cell survival	VGLL4-mediated inhibition of cIAPs	(32)
	Breast tumor	Repression of cell proliferation	VGLL4-mediated inhibition of STAT3	(33)
	Colorectal tumor	Repression of cell proliferation	VGLL4-mediated inhibition of TCF4-TEAD4 and WNT/b-catenin signaling	(34)
	Esophageal squamous cell carcinoma	Repression of cell proliferation and motility	VGLL4-mediated inhibition of CTGF expression	(35)
	Gastric tumor	Repression of cell proliferation	VGLL4-mediated inhibition of YAP-TEADs	(36)
	Gastric tumor	Repression of EMT	VGLL4-mediated inhibition of nuclear accumulation of b-catenin	(37)
	Gastric tumor	Repression of cell proliferation	IRF2BP2-mediated repression of VGLL4	(38)
	Gastric tumor	Repression of cell proliferation	miR-222-mediated VGLL4 repression	(39)
	Hepatocellular carcinoma	Negative corrilation with poor prognosis	miR-301a-3p-mediated VGLL4 repression	(40)
	Liver tumor	Repression of cell proliferation	IRF2BP2-mediated stabilization of VGLL4	(41)
	Lung tumor	Repression of cell proliferation	VGLL4-mediated inhibition of YAP-TEADs	(42)
	Lung tumor	Induction of tumor immune ivasion	VGLL4-mediated PD-L1 expression	(43)
	Pancreatic tumor	Repression of cell proliferation	CDK1-mediated phosphorylation and inhibition of VGLL4	(8)
	Renal cell adenocarcinoma	Repression of cell proliferation and invasion	USP11-mediated deubiquitination and stabilization of VGLL4	(44)
	Murine Liver tumor	Tumorigenesis and size control	miR-130a-mediated VGLL4 repression	(45)
	Murine pancreatic adenocarcinoma	Tumor suppressor gene	Enhancement of tumor progression by VGLL4 inactivation	(46)

## VGLL1 Is Involved in Tumor Progression

VGLL1 (TONDU) was the first identified human ortholog of Vg which could substitute for Vg in wing formation in *Drosophila* (6). The structural analysis of VGLL1-TEAD4 complex revealed that VGLL1 interacts with the surface of TEAD that overlap with YAP/TAZ-binding sites despite having a varied primary sequence and that VGLL1 competes with YAP/TAZ for TEAD binding (22). VGLL1 was detected in human prostate tumor cell lines PC3 and LnCAP, and stable expression of VGLL1 enhanced anchorage-independent growth on soft agar. VGLL1-expressing cells showed enhanced expression of insulin-like growth factor binding protein-5 (IGFBP5), a cell growth-promoting gene, whereas this enhancement was not observed with the overexpression of TAZ. Importantly, anchorage-independent growth or IGFBP5 expression was not induced by stable expression of the VGLL1 mutant lacking TEAD-binding ability, suggesting that VGLL1 depends on TEAD for its oncogenic activity (22).

Recently, *EWSR1-VGLL1* fusion genes were found in a soft tissue malignant myoepithelial tumor and a pediatric neuroepithelial neoplasm (21, 23). In each case, the fusion gene encodes a protein where the N-terminal transactivation domain of EWSR1 is fused to the full-length VGLL1. Although the exact oncogenic effect of *EWSR1-VGLL1* is still obscure, given the oncogenic roles of EWSR1 fusion genes, such as *EWSR1-FLI1* and *EWSR1-ERG*, in Ewing sarcoma (47), *EWSR1-VGLL1* likely has a potential to act as an oncogene.

The expression of VGLL1 was detected in fetal human lung and kidney (6). The analysis of VGLL1 expression in human breast tumor revealed that increased expression of VGLL1 is often detected in malignant types of breast tumor (triple negative and basal-like) and that VGLL1 expression is associated with reduced overall survival. It has been suggested that the modulation of estrogen receptor (ER) is involved in increased VGLL1 expression (18). Increased expression of VGLL1 has also been reported in gastric tumor, and its correlation with phosphatidylinositol-3-phosphate kinases (PI3K)/AKT/β-catenin signaling has also been demonstrated. VGLL1 is required for gastric tumor cell growth and metastasis, and matrix metalloprotease 9 (MMP9) has been suggested to be a target of VGLL1-TEAD4 complex (20).

Because TEAD1 activates the early promoter of human papillomavirus (HPV), a causative agent for cervical tumor, the contribution of VGLL1 to HPV early gene expression was recently investigated (19). The knockdown of VGLL1 reduced viral early gene expression in human cervical keratinocytes and cervical cancer cell lines. VGLL1 bound the HPV16 long control region (LCR) as a VGLL1-TEAD1 complex. The introduction of HPV16 and HPV18 whole-genomes into primary human keratinocytes increased VGLL1 expression. The results of these studies suggested that the VGLL1-TEAD1 complex support efficient transcription of HPV early genes, following cervical tumor development (Figure 1B).

## VGLL2 Is A Target of Gene Alteration in SARCOMA

VGLL2, also known as vestigial and TONDU related (VITO)-1, was identified as a VGLL1 homolog specifically expressed in the skeletal muscle lineage (48, 49). VGLL2 association with TEAD1 and VGLL2 overexpression enhanced the induction of myosin heavy chain, a marker of terminal differentiation of muscle. VGLL2-knockout mice showed an increased number of fast-twich type IIb fibers and a down-regulation of slow type I myosin heavy chain gene. These knockout mice exhibited exercise intolerance, suggesting that VGLL2 is involved in the differentiation of the muscle (50, 51).

Two kinds of *VGLL2*-fusion genes, *VGLL2-CITED2* and *VGLL2-NCOA2*, were identified in pediatric spindle and sclerosing rhabdomyosarcoma (SRMS) (24). SRMS is a type of muscle tumor that occurs in very young children. Each *VGLL2*-fusion gene encodes a protein where the C-terminal region of VGLL2 was replaced by CITED2 or NCOA2 gene product. Sarcomas harboring *VGLL2*-fusion genes shared similar gene expression signatures (52), suggesting that the common region of these fusion genes, namely VGLL2, plays an important role in the development of sarcoma. The analyses of the molecular roles of *VGLL2*-fusion genes are required to evaluate their significance in sarcoma development (Figure 1C).

## VGLL3 Is Involved in Both Tumor Development and Suppression

VGLL3 was found as a VGLL1 homolog predominantly expressed in the placenta (48). VGLL3 was also identified as VITO-2, which shares a high homology with VITO-1 and is mainly expressed in the myogenic lineage during early mouse embryonic development (53). In adult mice, VGLL3 was detected in various tissues, including the skeletal muscle, heart, kidney, liver, and brain (53). Mammalian two-hybrid assays showed the association between VGLL3 and TEAD1 (54). RNA interference-mediated VGLL3 knockdown suppressed myoblast proliferation, and VGLL3 overexpression strongly promoted myogenic differentiation (55). These observations suggested that VGLL3-TEAD1 complex regulates the differentiation of various types of cells, including muscles.

Similarly to *VGLL2, VGLL3* gene alterations were identified in sarcoma. *VGLL3* gene amplification and overexpression were found in myxoinflammatory fibroblastic sarcoma and soft tissue sarcoma (27, 29). Knockdown experiments showed that VGLL3 is required for proliferation in a soft tissue sarcoma-derived cell line (29). Recent deep sequencing of myxoinflammatory fibroblastic sarcoma demonstrated that *VGLL3* amplification is a highly recurrent feature of this type of sarcoma (56).

In addition to sarcoma, *VGLL3* expression was found to be positively correlated with accelerated grade and poor prognosis in gastric tumor (26). VGLL3-high gastric tumor showed the activation of the MAPK, JAK-STAT, and WNT pathways together with enhanced immune infiltrates (57). These features in VGLL3-high tumors may reflect the proinflammatory functions of VGLL3 which were found in VGLL3-overexpressing mice (58). Transforming growth factor-β (TGF-β) induced VGLL3 expression in a histone modification-dependent manner (59). Therefore, VGLL3 may be involved in TGF-β-related cell responses, such as epithelial-to-mesenchymal transition (EMT), in VGLL3-amplified or -high tumor cells (Figure 1D).

Recently, VGLL3 was found to promote proliferation of breast tumor and sarcoma cells by inducing LATS2 expression and Hippo pathway activation, suggesting that the Hippo pathway promotes tumor cell proliferation through inhibition of YAP/TAZ in the presence of VGLL3 (25). Notably, YAP/TAZ are known to function as a tumor suppressor via a cell-autonomous mechanism (60–62) and a non-cell-autonomous mechanism (63). Therefore, relationship between VGLL3-dependent cell growth and the tumor suppressive role of YAP/TAZ needs to be evaluated.

The tumor suppressor role of VGLL3 was also suggested in ovarian tumor. The transfer of a chromosome 3 fragment containing *VGLL3* gene suppressed tumor phenotypes in the ovarian tumor cell line OV90 (28). VGLL3 expression in parental OV90 cells was undetectable, and the transfer of the chromosome fragment rescued VGLL3 expression and repressed tumorigenicity, suggesting that VGLL3 is a tumor suppressor gene (64). However, VGLL3 single gene transfer did not cause significant reduction in the proliferation of OV90 cells *in vitro* and *in vivo* (65). The concept of the tumor-suppressing role of VGLL3 needs more evaluation.

## VGLL4 Is A Tumor Suppressor in Various Types of Tumor

VGLL4 is the only member of VGLL expressed in the heart (66). Unlike other members of VGLL that have a single TDU motif, VGLL4 has two tandem TDU motifs in its C-terminal region. VGLL4 association with TEAD1 and the overexpression of VGLL4 in cardiac myocytes repressed TEAD1-dependent skeletal α-actin promoter activity, suggesting that VGLL4 is a negative regulator of TEAD1 (66).

Consistent with this repressive effect of VGLL4 on TEAD activity, *VGLL4* is recognized as a tumor suppressor gene (67). The tumor-suppressing role of VGLL4 was first observed in the transposon *Sleeping Beauty*-mediated mutagenesis in murine *Kras*-driven pancreatic adenocarcinoma models (46). The reduction in VGLL4 expression was observed in human lung tumor, and VGLL4 expression repressed the proliferation of lung tumor cells via the suppression of TEAD transcriptional activities (42). VGLL4 was down-regulated in esophageal squamous cell carcinoma, which led to increased cell growth and motility through the induction of the expression of connective tissue growth factor (CTGF) (35). Low expression of VGLL4 positively correlated with poor prognosis of gastric tumor patients, and reduction in VGLL4 expression increased YAP-mediated TEAD activity and gastric tumor cell growth (36, 37). Mechanistically, VGLL4 directly competed with YAP for TEAD binding. Structural and biochemical analyses revealed that the tandem TDU motifs in VGLL4 are not only essential but also sufficient for its suppressive role on YAP (36). VGLL4 repressed the proliferation of breast tumor cells via the inhibition of YAP-mediated gene induction, and high expression of VGLL4 correlated with poor prognosis of breast tumor patients (31).

Although the tumor-suppressing roles of VGLL4 mostly depend on competition with YAP for TEAD binding, VGLL4 also acts as a tumor suppressor in a YAP-independent manner. VGLL4 bound and inhibited cellular inhibitor of apoptosis proteins (cIAPs) and consequently promoted apoptotic cell death (32). VGLL4 associates with T-cell factor 4 (TCF4), a transcription factor in WNT/β-catenin signaling, and interferes with the formation of TCF4-TEAD4 complex. This VGLL4-mediated inhibition of TCF4-TEAD4 formation repressed WNT/β-catenin signaling and colorectal cancer progression (34). VGLL4 also suppresses EMT in gastric tumors by inhibiting WNT/β-catenin signaling via repression of the nuclear accumulation of β-catenin and activation of TCF/LEF target genes (37, 67). STAT3, a transcription factor in JAK-STAT signaling, was another target of VGLL4, and binding of VGLL4 to STAT3 repressed its transcriptional activity and cell growth in triple-negative breast cancer (33).

What are the molecular mechanisms of VGLL4 down-regulation in tumor cells? MicroRNAs are involved in VGLL4 repression. MiR-222 repressed VGLL4 expression and in turn activated YAP-TEAD signaling and cell growth in gastric tumor cells (39). MiR-130a, which is a direct target of YAP-TEAD complex, repressed VGLL4 expression and thereby amplified YAP-TEAD activity. This miR-130a-mediated repression of VGLL4 was involved in murine liver tumorigenesis and size control (45). MiR-301a-3p, which repressed VGLL4 expression, was enhanced in human hepatocellular carcinoma tissues and cell lines, and higher miR-301a-3p expression showed positive correlation with poor prognosis in tumor patients (40). MiR-130b was up-regulated in bladder tumor and promoted proliferation, migration, and invasion of bladder tumor cell lines via the repression of VGLL4 (30).

MicroRNA-independent mechanisms of VGLL4 repression in tumor have also been reported. Cyclin-dependent kinase1 (CDK1)-mediated phosphorylation suppressed the tumor-suppressing activity of VGLL4 (8). Serotonin 5-hydroxytryptamine could control YAP/VGLL4 balance and promote hepatocellular carcinoma progression (68). Hypoxic stress, which is a frequently observed characteristic in tumor, caused alternative splicing of VGLL4 gene in human breast tumor cells, and this alternative splicing was suggested to affect its tumor-suppressing role (69). Ubiquitin-specific protease 11 (USP11) deubiquitinated and stabilized VGLL4 proteins, and the inactivation of USP11 was suggested to be involved in the destabilization of VGLL4 in tumor cells (44).

Although interferon regulatory factor 2 binding protein 2 (IRF2BP2) was identified as a VGLL4 binding partner (70), the relationship between these proteins are complicated. IRF2BP2 stabilized VGLL4 protein and repressed tumor progression via the inactivation of YAP-TEAD4 complex in liver cancer (41). In contrast, IRF2BP2 repressed the suppressive role of VGLL4 on YAP-TEAD activation and promoted cell growth by inducing CTGF expression in gastric cancer (38). It has also been reported that IRF2BP2 and VGLL4 promote tumor growth through the induction of the immune checkpoint protein programmed cell death-ligand 1 (PD-L1) and immune evasion of tumor cells (43). Furthermore, VGLL4 was shown to act as a positive regulator for TEADs together with IRF2BP2 and promote expression of the angiogenic factor vascular endothelial growth factor A (VEGFA), suggesting that VGLL4 has a potential to activate TEADs in the presence of IRF2BP2 (70). The relationship between IRF2BP2 and VGLL4 is likely to be determined by cell context, and more detailed analyses are required (Figure 1E).

## Conclusions

VGLL1 dysregulation was detected in various types of tumor; however, gene alterations in VGLL2 and VGLL3 were observed specifically in sarcoma. VGLL2 shares a high homology with VGLL3, and both genes are expressed in the myogenic lineage (53). Recent studies revealed that VGLL2 and VGLL3 are involved in the differentiation of muscle cells (51, 55). Therefore, the alterations of each gene are likely to affect proliferation and differentiation of stem cells in the myogenic lineage. VGLL2 and VGLL3 may be myogenic lineage-specific oncogenes (66), and this hypothesis should be evaluated in the future.

The inactivation of VGLL4 is involved in various types of tumors. VGLL4 expression has been observed in a wide range of tissues (66), and hence, it is likely to be a ubiquitously expressed tumor suppressor. Therefore, the transfer of VGLL4 into tumor cells may be an effective therapeutic method. Actually, adenovirus-mediated transfer of VGLL4 into hepatocellular carcinoma cells selectively killed the tumor cells through cell cycle arrest and apoptosis induction (71). On the basis of the structural and biochemical analyses of VGLL4-TEAD complex, Jiao and colleagues developed a VGLL4-mimicking peptide that acts as a YAP antagonist (36). The administration of this peptide significantly repressed YAP activation and gastric tumor growth, indicating that the targeting of YAP/TAZ-TEAD complex by the VGLL4-mimicking peptide is a promising therapeutic strategy for various tumors.

Because VGLL1-3 are suggested to be involved in tumor progression, its inactivation is required for tumor treatment. However, upstream signal transduction pathways controlling VGLL1-3 remain largely unknown. Post-transcriptional modifications (PTMs), such as protein phosphorylation, are key molecular mechanisms that regulate protein complex formation, subcellular localization, and stability. The PTMs of VGLL4 were reported (Figure 1A): CDK1 phosphorylates VGLL4 and lowers its affinity to TEADs (8), and the histone acetyltransferase p300 acetylates the lysine residue in the first TDU motif of VGLL4 and suppress its association with TEADs (9). Similarly to VGLL4, PTMs is likely to regulate binding of VGLL1-3 to TEADs. Identification of PTMs that control association of VGLL1-3 with TEADs and development of methods that could specifically repress this complex formation is required for tumor treatment.

In *Xenopus laevis*, VGLL3 binds to ETS-1, a transcription factor other than TEADs, and regulates trigeminal nerve formation and cranial neural crest migration (72). Given that ETS-1 plays an oncogenic role in various tumors (73) and that VGLL4 regulates transcription factors other than TEADs, it is reasonable to hypothesize that VGLL3 cooperates with ETS-1 as well as TEADs to promote tumorigenesis. Understanding of the whole picture of the binding targets of VGLL members might be helpful in understanding the complexity of the role of VGLL members in tumor development.

To estimate side-effects of molecular targeted therapies, understanding of the phenotypes of knockout mice is useful. Because VGLL4 knockout mice show severe defects in heart valve development and homeostasis (74), VGLL4-tageted drugs may have a risk to affect the development and homeostasis of heart. In contrast, VGLL2 or VGLL3 single knockout mice show only slight abnormalities in skeletal muscle (50, 51, 55). Therefore, VGLL2- or VGLL3-targeted drugs may be preferable medications for tumors with low risk of side-effects.

## Author Contributions

The author confirms being the sole contributor of this work and has approved it for publication.

## Conflict of Interest

The author declares that the research was conducted in the absence of any commercial or financial relationships that could be construed as a potential conflict of interest.
